# 1295. Impact of Inoculum Size on Clinical Presentation in a Murine Knee Arthroplasty Model of Periprosthetic Joint Infection

**DOI:** 10.1093/ofid/ofad500.1134

**Published:** 2023-11-27

**Authors:** Melissa J Karau, Diego Alarcon Perico, Judith Alvarez Ortero, Christina Koscianski, Robin Patel, Nicholas Bedard

**Affiliations:** Mayo Clinic, Rochester, Minnesota; Mayo Clinic, Rochester, Minnesota; Mayo Clinic, Rochester, Minnesota; Mayo Clinic, Rochester, Minnesota; Mayo Clinic, Rochester, Minnesota; Mayo Clinic, Rochester, Minnesota

## Abstract

**Background:**

Periprosthetic joint infection (PJI) is a devastating complication following joint replacement, associated with morbidity and mortality. Here, we established a clinically representative murine model of knee arthroplasty and determined the ideal bacterial inoculum that would result in PJI without causing significant limb compromise.

**Figure 1.**
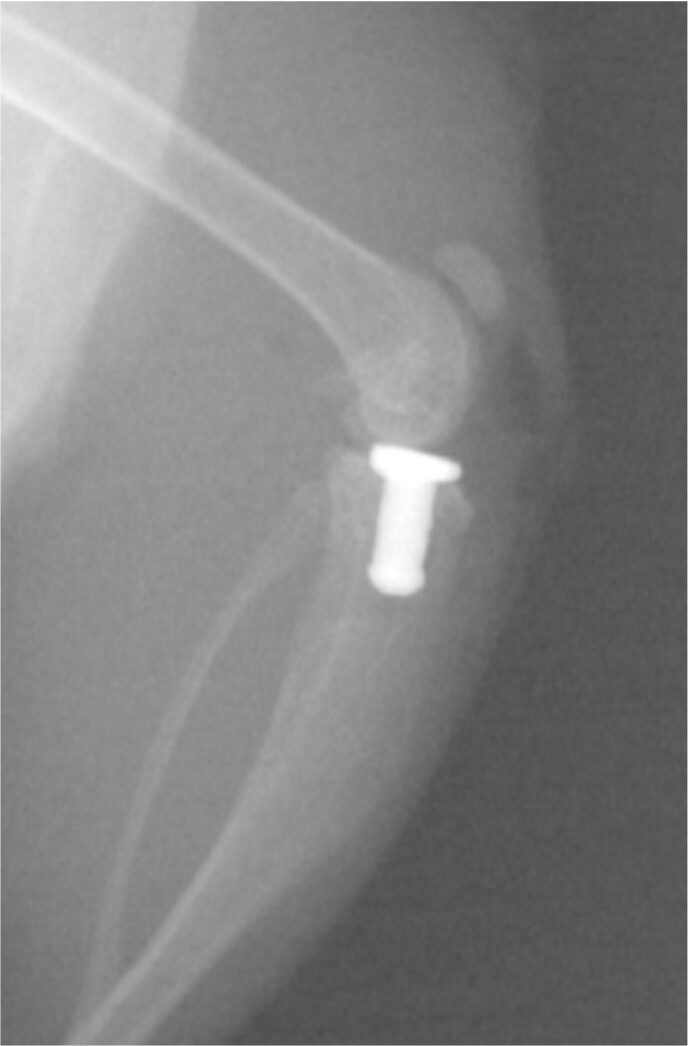
An example of an uninfected titanium prosthesis osseointegrated into the tibia of a mouse.

**Methods:**

A titanium tibial prosthesis designed for the murine knee was 3D printed and surgically implanted into the proximal tibia (Figure 1) of 38 C57BL/6 mice. The prosthesis is designed to osseointegrate into the tibia and has a smooth articular surface to allow for immediate weight bearing and range of motion. At the time of surgery, the arthroplasty was inoculated with five inocula of *Staphylococcus aureus* Xen36, ranging from 29 to 14,000 colony forming units (CFU) (Table 1). Mice were sacrificed at 3 weeks, or earlier if necessary due to infectious sequelae, and the joint tissue, tibia, femur, and prosthesis quantitatively cultured.

**Results:**

All mice inoculated with 181 to 14,000 CFU developed PJI as did 90% inoculated with 29 CFU. Bacterial loads from joint tissues and tibial prostheses are shown in Table 1. All mice inoculated with 14,000 and 760 CFU had overt findings of infection (abscesses, sinus tracts, significant limb dysfunction) requiring early euthanasia (Table 2). Mice inoculated with 640 CFU had swelling, redness, and small sores in first 3 days, and 1 death, but otherwise appeared well; only 2/9 had a visible abscess at 3-week sacrifice. 2/10 mice receiving 181 CFU had small sores that resolved in the first week; all limbs functioned well prior to sacrifice. Mice receiving 29 CFU had no external evidence of infection, however 9/10 had purulence within the joint at sacrifice.

**Figure:**
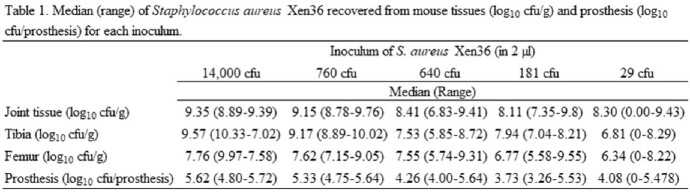

**Figure:**
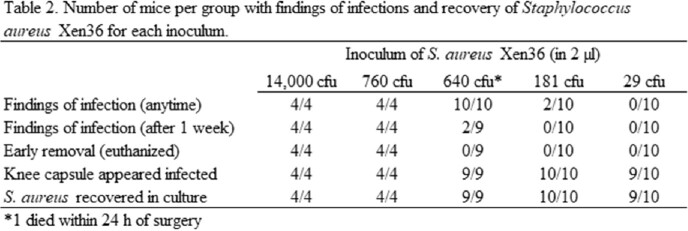

**Conclusion:**

This study demonstrates that inoculum size directly impacts clinical manifestations of infection in a murine model of *S. aureus* PJI. An inoculum of 181 CFU resulted in universal infection without significantly compromising the limb. The ability of this model to create PJI without overt infectious sequela will allow for evaluation of therapeutics, including those locally delivered.

**Disclosures:**

**Robin Patel, MD**, Abbott Laboratories: Advisor/Consultant|Adaptive Phage Therapeutics: Grant/Research Support|Adaptive Phage Therapeutics: Mayo Clinic has a royalty-bearing know-how agreement and equity in Adaptive Phage Therapeutics.|BIOFIRE: Grant/Research Support|CARB-X: Advisor/Consultant|ContraFect: Grant/Research Support|Day Zero Diagnostics: Advisor/Consultant|HealthTrackRx: Advisor/Consultant|Mammoth Biosciences: Advisor/Consultant|Netflix: Advisor/Consultant|Oxford Nanopore Technologies: Advisor/Consultant|PhAST: Advisor/Consultant|See details: Patent on Bordetella pertussis/parapertussis PCR issued, a patent on a device/method for sonication with royalties paid by Samsung to Mayo Clinic|See details: continued, patent on an anti-biofilm substance issued|TenNor Therapeutics Limited: Grant/Research Support|Torus Biosystems: Advisor/Consultant|Trellis Bioscience, Inc.: Advisor/Consultant **Nicholas Bedard, MD**, Stryker: Advisor/Consultant

